# Cryptochromes integrate green light signals into the circadian system

**DOI:** 10.1111/pce.13643

**Published:** 2019-08-27

**Authors:** Martin William Battle, Matthew Alan Jones

**Affiliations:** ^1^ School of Life Sciences University of Essex Colchester CO4 3SQ UK; ^2^ Institute of Molecular, Cell and Systems Biology University of Glasgow Glasgow G12 8QQ UK

**Keywords:** circadian, cryptochromes, light quality, photobiology, signalling

## Abstract

Plants are acutely sensitive of their light environment, adapting their growth habit and prioritizing developmental decisions to maximize fecundity. In addition to providing an energy source and directional information, light quality also contributes to entrainment of the circadian system, an endogenous timing mechanism that integrates endogenous and environmental signalling cues to promote growth. Whereas plants' perception of red and blue portions of the spectrum are well defined, green light sensitivity remains enigmatic. In this study, we show that low fluence rates of green light are sufficient to entrain and maintain circadian rhythms in Arabidopsis and that cryptochromes contribute to this response. Importantly, green light responses are distinguishable from low blue light‐induced phenotypes. These data suggest a distinct signalling mechanism enables entrainment of the circadian system in green light‐enriched environments, such as those found in undergrowth and in densely planted monoculture.

## 1 INTRODUCTION

Plants are highly sensitive to changes in ambient light conditions, with a complex photosensory network evolving to facilitate the cell‐autonomous perception of light across the electromagnetic spectrum. In addition to being used as an energy source, plants decipher the composition and duration of light perceived to make appropriate developmental decisions. Whereas photomorphogenesis is a critical component of early development, light also informs development in mature tissues (Whitelam & Halliday, 2007). In general, red and blue light enable plants to orientate themselves appropriately within a canopy, whereas far‐red and green‐enriched light is perceived as an indication of overgrowing vegetation, inducing a shade avoidance response (Casal, 2013; Liscum et al., 2014; Wang, Zhang, & Folta, 2015; Zhang & Folta, 2012). In combination, these responses allow plants to optimize their light‐gathering capacity.

Prior plant biology literature has subdivided the light spectrum into ultraviolet (UV) (320–400 nm), blue (400–500 nm), green (500–600 nm), red (600–700 nm), and far‐red portions (700–800 nm). Although UV, blue, and red/far‐red photoreceptors have been identified and well characterized, specific green photoreceptors have yet to be identified in higher plants (Christie, Blackwood, Petersen, & Sullivan, 2015; Rizzini et al., 2011; Wang & Wang, 2015). Instead, our current understanding suggests that plants perceive green light through the residual sensitivity of blue and red photoreceptors, with phytochromes and cryptochromes absorbing portions of the green spectrum, albeit at a fraction of the sensitivity towards their primary wavelength (Sellaro et al., 2010; Smith, McAusland, & Murchie, 2017; Wang, Maruhnich, Mageroy, Justice, & Folta, 2013). In addition, plants are able to harvest green light for photosynthesis via both chlorophylls and carotenoids (Smith et al., 2017), whereas a distinct role for zeaxanthin as a blue/green reversible photoreceptive pigment in guard cells has also been proposed (Frechilla, Talbott, Bogomolni, & Zeiger, 2000; Talbott et al., 2006; Talbott, Zhu, Han, & Zeiger, 2002). Seedlings' perception of blue:green ratios regulates hypocotyl extension, suggesting that plants interpret blue:green ratios as a shade response, whereas green light has also been reported to antagonize blue‐ and UV‐B induced stomatal opening (Casal, 2012; Eisinger, Bogomolni, & Taiz, 2003; Sellaro et al., 2010; Smith et al., 2017; Talbott et al., 2006). These reports highlight the importance of understanding how plants respond to complex, multichromatic lighting regimes.

Cryptochromes have previously been reported to perceive both blue and green portions of the spectrum (Ahmad et al., 2002; Lin, Ahmad, Gordon, & Cashmore, 1995). Although dark‐adapted cryptochromes do not absorb light wavelengths longer than 500 nm, illuminated cryptochrome photocycle intermediates absorb light up to 650 nm (Banerjee et al., 2007; Bouly et al., 2007). It has subsequently been proposed that shorter wavelengths of green light (<530 nm) are perceived as part of the canonical cryptochrome and phototropin blue light response, whereas longer wavelengths of green/yellow light (~570 nm) accelerate the reversion of blue‐light activated cryptochrome to its inactive state (Bouly et al., 2007). This latter hypothesis provides an elegant photochemical explanation for the observed antagonization of blue photoperception by longer wavelengths of “green” light, although the photochemical mechanism underlying this remains elusive (Banerjee et al., 2007; Bouly et al., 2007; Herbel et al., 2013; Wang et al., 2013).

In addition to its role in development and photosynthesis, light quality and intensity also informs progression of the circadian system, an endogenous timing mechanism that coordinates metabolism, physiology, and development with prevailing environmental conditions. The pace, phase, and amplitude of the central oscillator are regulated by the quality and intensity of light irradiation, with both photoreceptors and photoassimilates contributing to the maintenance of circadian rhythms (Baudry et al., 2010; Devlin & Kay, 2000; Haydon, Mielczarek, Robertson, Hubbard, & Webb, 2013; Somers, Devlin, & Kay, 1998). Genes such as *CCA1* and *PRR9* are induced by light, whereas PRR7 and GIGANTEA monitor photoassimilate accumulation and modulate circadian timing accordingly (Haydon et al., 2013; Haydon, Mielczarek, Frank, Román, & Webb, 2017; Ito et al., 2003; Locke et al., 2005; Wang & Tobin, 1998). As a consequence, the circadian system provides a well understood readout of plant photoperception in addition to its role as a governor of plant development.

Here, we examine the role of cryptochromes as blue/green photoreceptors within the circadian system. We demonstrate that both cry1 and cry2 contribute to the clock's response to green light. Our data also distinguish between blue and green light‐induced phenotypes, suggesting that green light sensitivity is not merely a consequence of residual chromophore absorption above 500 nm. Finally, we determine that *cry* mutants continue to have a phenotype under blue/green light, suggesting shorter wavelengths of green light are insufficient to impair cry‐dependent blue light signalling into the circadian system.

## 2 MATERIALS AND METHODS

### 2.1 Plant materials and growth conditions

**Figure 1:**
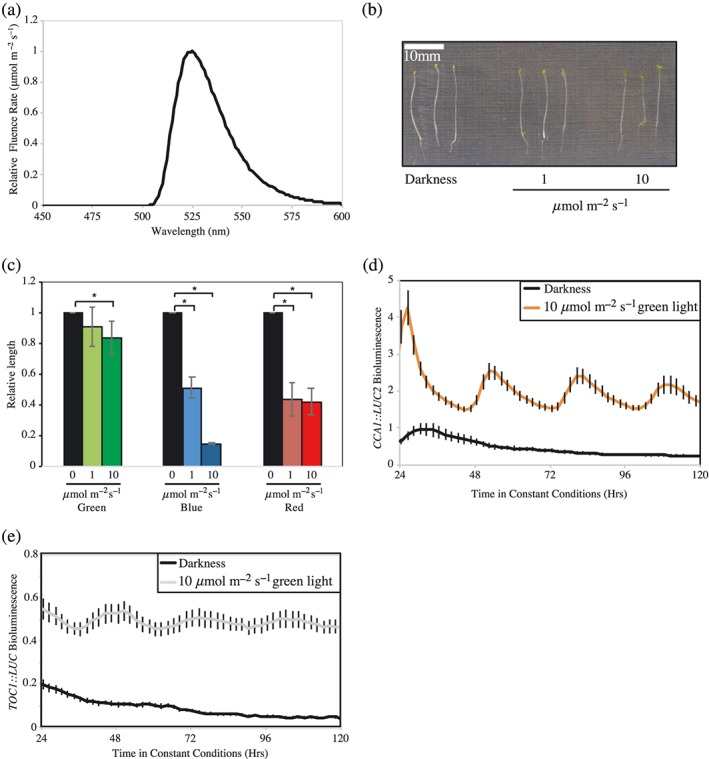
Characterization of plant responses to green light. (a) Spectrum of green light‐emitting diodes (LEDs) used in this study. (b) Representative image of wild type Columbia (Col) grown for five days in either darkness, or under 1 μmol m^−2^ s^−1^ or 10 μmol m^−2^ s^−1^ of green light. (c) Quantification of hypocotyl length measurements, relative to dark grown seedlings, of plants grown under either 1 μmol m^−2^ s^−1^ or 10 μmol m^−2^ s^−1^ of green, blue, or red light. Data were averaged from four independent experiments, *n* > 80. (d) Waveforms of luciferase bioluminescence rhythms of wild type *CCA1::LUC2* seedlings entrained for 6 days under 12 hr:12 hr light:dark cycles before transfer to either darkness or 10 μmol m^−2^ s^−1^ constant green light. (e) Waveforms of luciferase bioluminescence rhythms of wild type *TOC1::LUC* seedlings treated as described in (d). Data are representative of three independent experiments, *n* > 20. Error bars indicate SEM [Colour figure can be viewed at http://wileyonlinelibrary.com]

Experiments were conducted in *Arabidopsis thaliana* (Columbia, RRID: SCR_004618). Arabidopsis seeds were surface sterilized and sown on soil or 0.8% agar plates containing half‐strength MS medium (Sigma‐Aldrich M5524). *CCR2::LUC* and *phyB‐*9 *CCR2::LUC* lines have previously been reported (Jones, Hu, Litthauer, Lagarias, & Harmer, 2015). A wild‐type Columbia line expressing *CCA1::LUC2* (Jones et al., 2015) was crossed with either *cry1‐304 cry2‐1* (Mockler, Guo, Yang, Duong, & Lin, 1999) or *phyA‐211* (Reed, Nagatani, Elich, Fagan, & Chory, 1994) to obtain *cry1‐304 CCA1::LUC2*, *cry2‐1 CCA1::LUC2*, *cry1‐304 cry2‐1 CCA1::LUC2*, and *phyA‐211 CCA1::LUC2* seedlings. Plants were entrained under 12‐hr‐white‐light/12‐hr‐dark cycles under 60 μmol m^−2^ s^−1^ before circadian imaging. Red (~600–700 nm, peaking at ~660 nm) and blue (~420–510 nm, peaking at ~450 nm) light was provided by light‐emitting diodes (LEDs; Bright Technology Industrial Ltd., Shenzhen City, China). Green light was provided by LEDs that were filtered through Schott OG515 glass producing a spectral range of ~500–600 nm peaking at ~530 nm (illustrated in Figure 1a).

### 2.2 Hypocotyl measurements

Seeds were irradiated with cool fluorescent white light at 60 μmol m^−2^ s^−1^ for 4 hr before being moved to coloured LEDs as per experimental requirements and grown vertically for 5 days before being imaged and processed using ImageJ (Schneider, Rasband, & Eliceiri, 2012). The length of hypocotyls was normalized to the average length of a dark‐grown control.

### 2.3 Luciferase imaging

Plants were entrained for 6 days in 12:12 L/D cycles under white light on MS medium without sucrose before being sprayed with 3‐mM D‐luciferin in 0.01% (v/v) Triton X‐100 as previously described (Litthauer, Battle, Lawson, & Jones, 2015). Experiments performed using CO_2_‐depleted air were completed as previously described (Kircher & Schopfer, 2012). In brief, 5‐g sodalime was added to a double‐sealed bag enclosing the petri plate on which seedlings had been sown immediately before circadian imaging. Imaging was completed over 5 days using an Andor iKon‐M CCD camera controlled by μManager (Edelstein, Amodaj, Hoover, Vale, & Stuurman, 2010) before data were processed using ImageJ (Schneider et al., 2012). Patterns of luciferase activity were fitted to cosine waves using fast Fourier transform–nonlinear least squares (FFT‐NLLS; Plautz et al., 1997, Zielinski, Moore, Troup, Halliday, & Millar, 2014) to estimate circadian period length. Relative amplitude error is a measure of rhythmic robustness, with a value of 0 indicating an exact fit to a cosine wave (Plautz et al., 1997).

### 2.4 Real‐time reverse transcription polymerase chain reaction

Following entrainment, plants were transferred to 20 μmol m^−2^ s^−1^ blue light or green light provided by LEDs. Tissue was harvested at the indicated time before RNA was isolated from 10 to 15 seedlings for each data point using Tri Reagent® according to the manufacturer's protocol (Sigma Aldrich, Dorset, UK, http://www.sigmaaldrich. com). Reverse transcription was performed using RevertAid reverse transcriptase following DNase treatment (Fisher Scientific, Loughborough, UK, http://www.fisher.co.uk). Real‐time reverse transcription polymerase chain reaction was performed using a BioRad CFX96 Real‐Time system. Samples were run in triplicate, with starting quantity estimated from critical thresholds using the standard curve of amplification. Data for each sample were normalized to *APX3*, *IPP2*, and *At1g11910* expression as internal controls as previously described (Nusinow et al., 2011). Primer sets used are described in Table S1.

## 3 RESULTS

### 3.1 Green light maintains circadian rhythmicity

Plants sense light via specific photoreceptors and also indirectly via the acquisition of photoassimilates from photosynthesis (Jones, 2017). The red light‐sensitive phytochromes, as well as the blue light‐sensitive cryptochrome and phototropin families have been well described (Christie et al., 2015; Rockwell, Su, & Lagarias, 2006), but previous studies have described only limited roles for green light in plant development (Wang et al., 2013). In our study, we used a combination of green LEDs and cut‐off filters to illuminate plants with broadband green light (500–600 nm, Figure 1a). As previously described, seedlings grown under green light are comparatively insensitive to this portion of the spectrum, with only modest reductions in hypocotyl extension observed with increasing fluence rates of green light (Figure 1b,c; Ahmad et al., 2002).

Although we did not observe significant differences in hypocotyl elongation in response to increasing green light, we were curious whether green light was sufficient to maintain circadian rhythms of gene expression. Transgenic Arabidopsis seedlings expressing a luciferase circadian reporter maintain circadian rhythms for multiple days when transferred to constant white, blue, or red light (Millar, Straume, Chory, Chua, & Kay, 1995). By contrast, in the absence of light, or under dim blue light (1 μmol m^−2^ s^−1^), circadian rhythms of luciferase bioluminescence dampened to apparent arrhythmia within 24 hr in the absence of sucrose (Haydon et al., 2017, Jones et al., 2015; Figure S1). To evaluate the role of green light in circadian rhythms, plants carrying a bioluminescent circadian reporter (*CCA1::LUC2* or *TOC1::LUC* [Jones et al., 2015]) were entrained to 12:12 light:dark cycles before being transferred to 10 μmol m^−2^ s^−1^ constant green light (Figure 1d,e). As for blue and red light, we observed that green light was sufficient to sustain circadian rhythms of luciferase activity with both reporters, with *τ* = 28.17 ± 0.26 hr with *CCA1::LUC2* or *τ* = 27.65 ± 0.40 hr in *TOC1::LUC* seedlings, respectively. Such data demonstrate that dim green light is sufficient to maintain circadian rhythms, despite not inhibiting hypocotyl elongation.

### 3.2 The circadian system is responsive to green light

**Figure 2:**
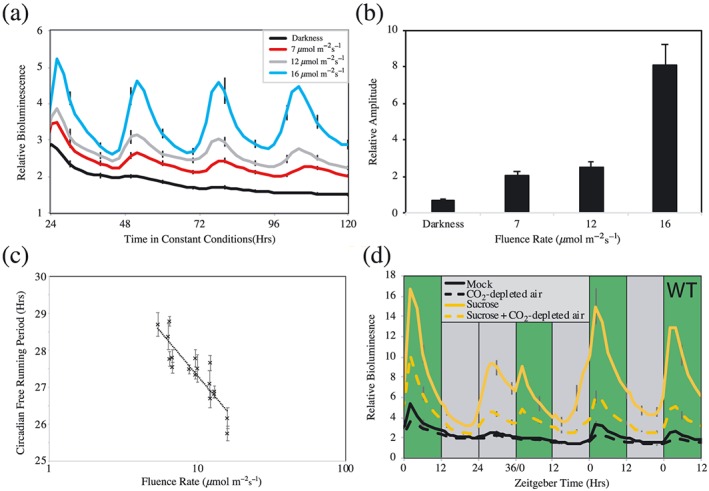
Effects of green light irradiation upon circadian rhythms in Arabidopsis. (a) Waveforms of luciferase bioluminescence rhythms of wild type *CCA1::LUC2* seedlings under either constant darkness or increasing fluence rates of constant green light. Plants were entrained under 12:12 light:dark cycles for 6 days before transfer to constant conditions with the indicated fluence rate of green light. (b) Relative amplitude of circadian rhythms of luciferase bioluminescence presented in (a). (c) Circadian free running period estimates of plants transferred to increasing fluence rates of constant green light. Plants were manipulated as described in (a). (d) Bioluminescence waveforms of wild type *CCA1::LUC2* seedlings imaged under diel cycles of 16 μmol m^−2^ s^−1^ green light. Plants were grown on either MS plates or MS plates supplemented with 3% (w/v) sucrose and transferred to a CO_2_ depleted atmosphere as indicated. An extended dark period was introduced on the second day of imaging to examine entrainment of the circadian system to green light irradiation. Green bands indicate periods of green light, whereas grey bands indicate periods of darkness. All data are representative of at least three independent experiments. Error bars indicate SEM and in (a) and (d) are presented once every 10 hr for clarity, *n* > 20 [Colour figure can be viewed at http://wileyonlinelibrary.com]

In order to better understand the effect of green light upon the circadian system, we completed a fluence rate response curve (Figure 2). As under blue or red light, the amplitude of *CCA1::LUC2* rhythms increased with fluence rate (*p* < .001, one‐way analysis of variance [ANOVA], Figure 2a,b; Jones et al., 2015). Similarly, the pace of the circadian system was accelerated as light intensity increased (*p* < .001, one‐way ANOVA), with circadian period decreasing to 25.74 ± 0.12 hr under 16 μmol m^−2^ s^−1^ green light (Figure 2c). Comparable data were observed with a *TOC1::LUC* reporter (Figure S2). We also examined whether green light was sufficient to entrain the circadian system (Figure 2d). Plants were entrained under white light before being transferred to alternating periods of 12 hr green light, 12 hr darkness. Following 24 hr under these conditions, dawn was delayed by 12 hr so that plants experienced an extended night. Plants treated in this way were able to entrain to the revised timing of dawn (Figures 2d and S2d), demonstrating that the circadian system is responsive to green light, either via a green photoreceptor or as a consequence of green light‐derived photosynthesis.

### 3.3 Photoactivated cryptochromes contribute to green light signalling into the circadian system

**Figure 3:**
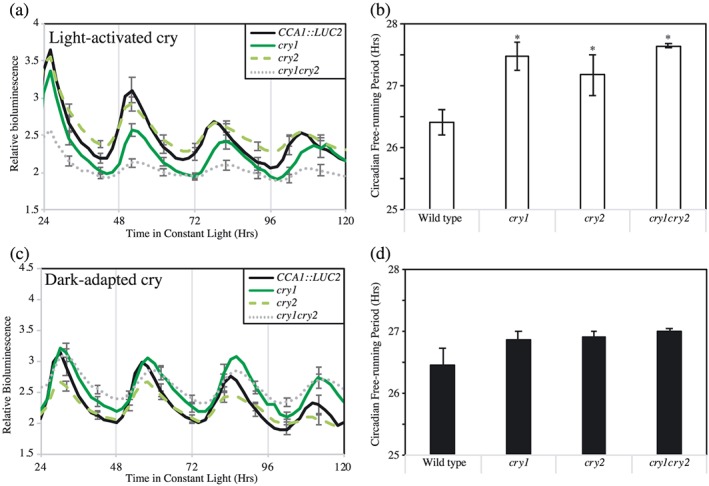
Assessment of circadian responses to green light in *cryptochrome* seedlings. (a) Luciferase bioluminescence of *CCA1::LUC2* plants monitored in continuous 16 μmol m^−2^ s^−1^ green light following direct transfer from entraining white light. Wild type, *cry1*, *cry2*, and *cry1cry2* seedlings were entrained for 6 days before being transferred to constant conditions at ZT10. (b) Circadian free running period of data presented in (a). (c) Luciferase bioluminescence of *CCA1::LUC2* plants monitored in continuous 16 μmol m^−2^ s^−1^ green light from dawn. Wild type, *cry1*, *cry2*, and *cry1cry2* seedlings were entrained for 6 days before being transferred to darkness at ZT12. Seedlings were moved to constant green light at ZT24 of Day 6. (d) Circadian free running period of data presented in (c). Error bars indicate SEM (*n* > 20) and, in (a) and (c), are presented every 10 hr for clarity. Asterisks indicate a significant difference from the applicable wild type control (*p* < .05, Dunnett's test) [Colour figure can be viewed at http://wileyonlinelibrary.com]

Of the known photoreceptors, cryptochromes have previously been described as blue/green photoreceptors, whereas phytochromes are also activated by green light (Lin et al., 1995; Shinomura et al., 1996). Although *phyA‐211* and *phyB‐9* mutants did not have a circadian phenotype under constant green light (Figure S3), we observed a significant extension of circadian period in *cryptochrome* mutants under these conditions (*p* < .01, Dunnett's test, Figure 3a,b). Under constant green light, *cry1* seedlings maintained a circadian rhythm with a period of 27.48 ± 0.23 hr, approximately 1 hr longer than wild type controls (Figure 3a,b, *τ* = 26.42 ± 0.20 hr). *cry2* seedlings similarly had an extended circadian period phenotype under these conditions (*τ* = 27.18 ± 0.33 hr), as did *cry1cry2* plants (*τ* = 27.65 ± 0.04 hr).

A comparison of absorbance spectra from dark‐adapted or illuminated cryptochromes indicate that these photoreceptors absorb proportionally more green light following illumination (Banerjee et al., 2007; Bouly et al., 2007). We were subsequently curious if *cry* seedlings transferred immediately to green light from the dark retained a circadian phenotype. The half‐life of photoactivated cryptochromes has been estimated to be approximately 6 min (Herbel et al., 2013), and so we transferred our plants into darkness for 12 hr (synchronized with the entraining night) before beginning our experiment under constant green light (Figure 3c,d). In contrast to plants transferred immediately from white light‐illuminated conditions (Figure 3a,b), we did not observe a significant difference in circadian period in *cry* plants illuminated solely with green light from dawn compared with wild type (*p* > .11, Figure 3c,d). These data suggest that photoactivated cryptochromes contribute to the maintenance of circadian rhythms under broadband green light.

### 3.4 Exogenous sucrose is sufficient to rescue the *cryptochrome* circadian phenotype under green light

**Figure 4:**
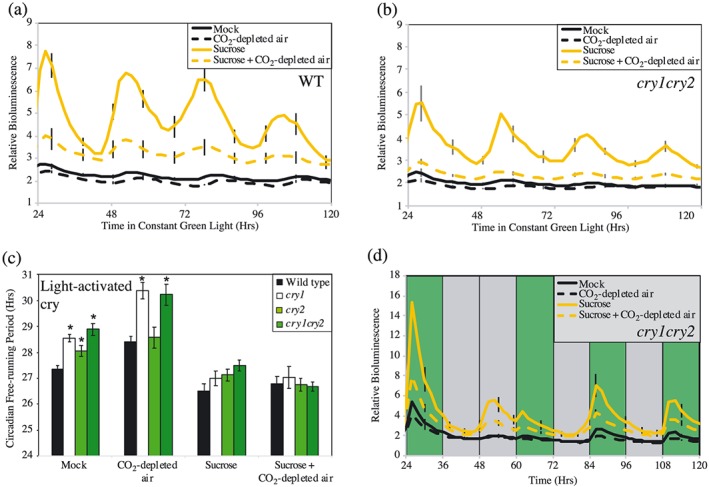
Determination of *cryptochrome* circadian phenotypes under constant green light in the presence of CO_2_‐depleted air or exogenous sucrose. (a,b) Waveforms of luciferase bioluminescence of wild type and *cry1cry2 CCA1::LUC2* seedlings imaged under constant green light in a CO_2_‐depleted environment. (c) Circadian free running period of plants transferred to 10 μmol m^−2^ s^−1^ constant green light in the presence of exogenous sucrose or in a CO_2_‐depleted atmosphere. (d) Bioluminescence waveforms of *cry1cry2 CCA1::LUC2* seedlings imaged under diel cycles of 16 μmol m^−2^ s^−1^ green light. An extended dark period was introduced on the second day of imaging to examine entrainment of the circadian system to green light irradiation. Green bands indicate periods of green light, whereas grey bands indicate periods of darkness. All data are representative of at least three independent experiments. Plants were grown on either MS plates or MS plates supplemented with 3% (w/v) sucrose [Colour figure can be viewed at http://wileyonlinelibrary.com]

Although our data suggest a role for cryptochromes in green light perception, recent work has emphasized the contribution of photoassimilates to circadian timing (Frank et al., 2018; Haydon et al., 2013; Haydon et al., 2017; Philippou, Ronald, Sanchez‐Villarreal, Davis, & Davis, 2019). In order to assess the contribution of photosynthesis towards circadian rhythmicity under constant green light, we assessed circadian rhythms in the presence of CO_2_‐depleted air, and/or by supplying exogenous sucrose within the growth media to saturate the cellular response to photosynthetically derived sucrose (Figures 2d and 4). As under constant dichromatic blue and red light (Haydon et al., 2013), or constant blue light (Figure S4), treatment with CO_2_‐depleted air reduced luciferase bioluminescence in plants transferred to constant green light, although there was no difference in circadian amplitude (Figure 4a,b). Indeed, CO_2_ depletion led to an extension of free‐running period (FRP) in each of the genotypes examined compared with a mock‐treated control under constant green light (Figure 4c). *Cry1* and *cry1cry2* plants maintained a long FRP under depleted CO_2_ conditions, although we found that the addition of sucrose to the growth media was able to rescue the circadian defect of *cry* plants under constant green light (Figure 4c). This suggests signalling pathways induced by exogenous sucrose are sufficient to mask the role of light‐adapted cryptochromes in the maintenance of circadian period (Figure 4c).

We next assessed whether CO_2_ depletion and exogenous sucrose were sufficient to limit entrainment of the circadian system to green light signals. Importantly, both wild type and *cry1cry2* plants subjected to an extended night in the presence of exogenous sucrose and CO_2_‐depleted air retained the ability to entrain via green light (Figures 2d and 4d). Such data suggest that although photosynthate‐derived signals contribute to the pace of the circadian oscillator under constant green light, photoreceptors retain an important role in circadian entrainment.

### 3.5 cry1 is required to maintain circadian amplitude under blue light

**Figure 5:**
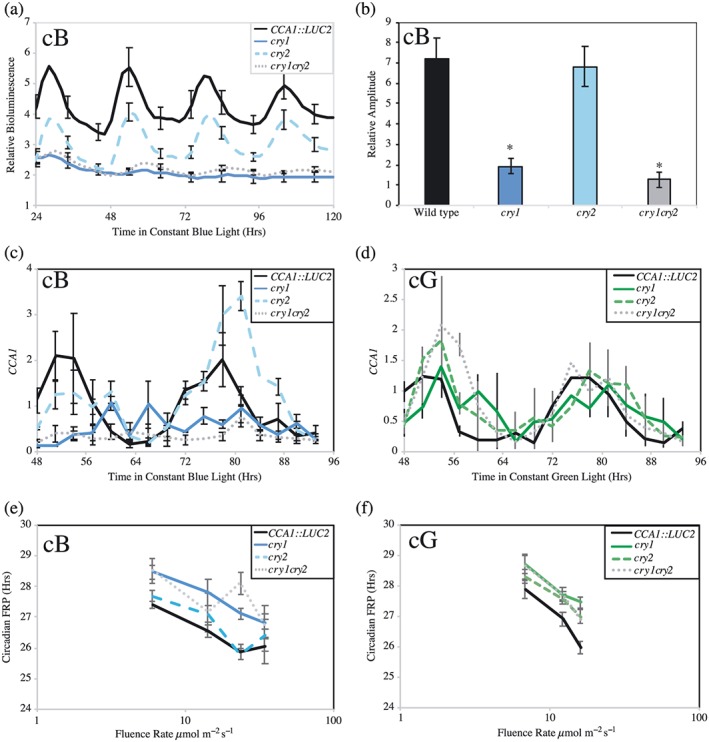
*CCA1* accumulation in *cryptochrome* seedlings under either blue and green light. Wild type, *CCA1::LUC2*, *cry1*, *cry2*, and *cry1cry2* seedlings were entrained for 6 days before transfer to constant conditions. (a) Waveforms of luciferase bioluminescence, imaged under 20 μmol m^−2^ s^−1^ constant blue light. Error bars indicate standard error of the mean and are shown every 10 hr for clarity (*n* > 10). (b) Relative amplitude of luciferase bioluminescence wavelengths presented in (a). Error bars indicate standard error of the mean (*n* > 10). (c,d) Real‐time reverse transcription polymerase chain reaction showing steady‐state accumulation of *CCA1* transcripts in wild type, *cry1*, *cry2*, and *cry1cry2* seedlings transferred to either 20 μmol m^−2^ s^−1^ constant blue light (c) or 20 μmol m^−2^ s^−1^ constant green light (d). Seedlings were grown under entraining conditions for 12 days before transfer to constant conditions. Data are the average of three independent experiments; standard error of the mean is shown. (e,f) Fluence rate response curves showing cryptochromes' role in blue light (e) and green light (f) input into the circadian system. Seedlings were entrained as described in (a) before being transferred to the indicated quality and quantity of light at ZT10. Error bars indicate standard error of the mean (*n* > 20) [Colour figure can be viewed at http://wileyonlinelibrary.com]

Given the epistatic role of exogenous sucrose in cryptochrome‐mediated circadian responses to green light (Figure 4c), we next sought to re‐examine the role of cryptochromes under blue light in the absence of exogenous sucrose (Figure 5). Interestingly, in contrast to plants grown in the presence of exogenous sucrose, we found that *cry1* plants had a greatly reduced circadian amplitude of *CCA1‐*driven bioluminescence in the absence of sucrose, which damped over circadian time (*p* < .001, Dunnett's test, Figures 5a,b, S4 and S5). These bioluminescence data were confirmed by real‐time reverse transcription polymerase chain reaction, with rhythmic *CCA1* and *GIGANTEA* transcript accumulation being greatly reduced in *cry1* and *cry1cry2* lines under constant blue light compared with wild type controls (Figures 5c and S6a). By contrast, the rhythmic amplitude of both *CCA1* and *GIGANTEA* expression was maintained in all genotypes when transferred to constant green light (Figures 5d and S6b).

We next completed fluence rate response curves to compare the effects of blue or green light on circadian period in light‐adapted *cryptochrome* seedlings. Under blue light, *cry1* seedlings had a significantly extended FRP under all fluence rates tested (~1 hr longer than WT plants, *p* < .001, ANOVA), whereas as previously reported, *cry2* lines were not distinguishable from wild type plants (Figure 5e, *p* = .067, ANOVA; Devlin & Kay, 1999, Somers et al., 1998). By contrast, under constant green light, the 1‐hr extension of FRP was observed in both light‐adapted *cry1* and *cry2* seedlings across a range of fluence rates (*p* < .001, ANOVA, Figures 3b and 5f). Interestingly, as under blue light, *cry1cry2* lines retained a fluence rate response to constant green light (*p* < .001, ANOVA). These data likely indicate the role of additional photoreceptors or photosynthates in the integration of green light into the circadian system.

### 3.6 Cryptochromes continue to signal into the circadian system in the presence of blue/green light

**Figure 6:**
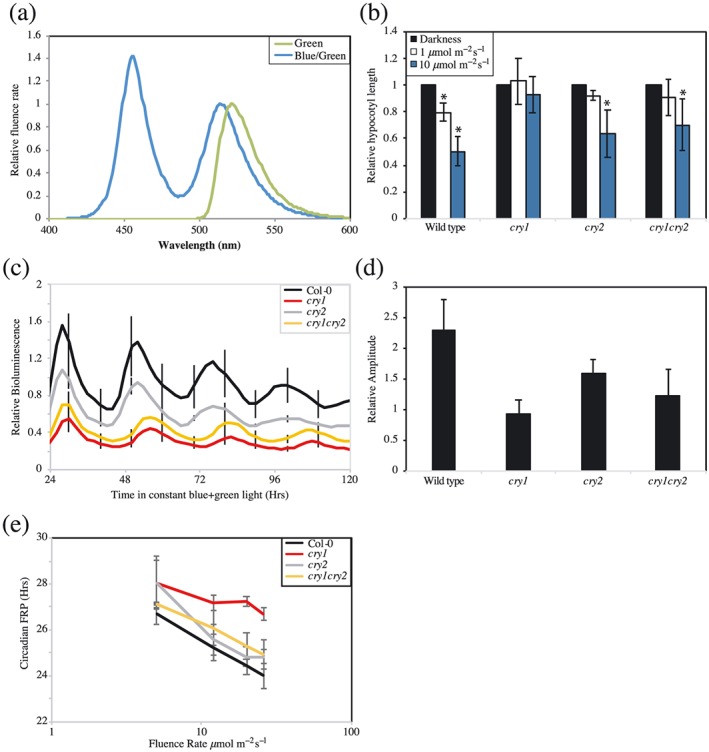
Assessment of circadian responses to blue/green light. (a) Spectra of green light and of blue/green light used. (b) Average hypocotyl length of wild type, *cry1*, *cry2*, and *cry1cry2* seedlings grown vertically for 5 days in constant darkness, 1 μmol m^−2^ s^−1^, or 10 μmol m^−2^ s^−1^ of constant blue/green light. Data were averaged from three independent experiments, *n* > 60. (c) Circadian rhythms of luciferase bioluminescence under blue/green light. Wild type, *cry1*, *cry2*, and *cry1cry2* seedlings were entrained for 6 days before transfer to 25 μmol m^−2^ s^−1^ constant blue:green light. Error bars indicate standard error of the mean and are shown every 10 hr for clarity (*n* > 10). (d) Relative amplitude of rhythmic luciferase bioluminescence presented in (c). (e) Fluence rate response curve showing cryptochromes' contribution to blue/green light input into the circadian system. Error bars show standard error of the mean (*n* > 20). All data are representative of at least three independent experiments [Colour figure can be viewed at http://wileyonlinelibrary.com]

Plants' perception of green light is particularly important under shaded canopies, with the ratio of blue:green light dropping to approximately 0.5 under deeply shaded canopies (Sellaro et al., 2010; Smith et al., 2017). As cryptochromes have been proposed to act as reversible blue/green photoreceptors (Banerjee et al., 2007; Bouly et al., 2007; Herbel et al., 2013; Sellaro et al., 2010), we examined the circadian phenotype of plants transferred to conditions comparable with deep shade to determine whether altering the blue/green ratio altered circadian timing (Figure 6). Hypocotyl elongation was inhibited in wild type plants under these blue/green conditions, although *cry1* plants were unresponsive, presumably because they are unable to perceive the blue light component of the light source (Figure 6a,b). Interestingly, circadian rhythms were maintained in these conditions in all genotypes, with *cry1* plants having a significantly extended circadian period compared with wild type controls (Figure 6c, *τ* = 26.68 ± 0.26 hr in *cry1* and *τ* = 23.99 ± 0.56 hr in wild type, *p* = .01, Dunnett's test). By contrast, although *cry2* seedlings had an extended circadian period under green light (Figure 5f), under blue/green light, these seedlings were indistinguishable from wild type (Figure 6e, *p* = .25). Such data suggest that cry1 contributes to the maintenance of circadian rhythms under conditions equivalent to deep shade.

## 4 DISCUSSION

### 4.1 Cryptochromes contribute to green light signalling into the circadian system

A specific green photoreceptor remains elusive in plants, although the role of green light as a regulator of plant development in response to shade has begun to emerge in recent years (Wang & Folta, 2013). Phytochromes, phototropins, and cryptochromes have each been implicated in specific green light responses ranging from hypocotyl growth inhibition to petiole elongation (Zhang, Maruhnich, & Folta, 2011). Our work with short wavelength green light reveals that green light is additionally sufficient to maintain circadian rhythms, despite this portion of the electromagnetic spectrum having little effect upon the inhibition of hypocotyl elongation (Figures 1 and 2).

Cryptochromes were originally identified as blue/green photoreceptors, although the effect of green light upon cryptochrome photoperception has proven to be complex. Plants overexpressing *CRY1* have previously been reported as being hypersensitive to short‐wavelength green light (<532 nm, Ahmad et al., 2002, Bouly et al., 2007) whereas long‐wavelength green light centered around 570 nm is sufficient to antagonize cryptochrome activation (Banerjee et al., 2007; Bouly et al., 2007; Herbel et al., 2013). Our experiments, utilizing a short‐wavelength green light (peak 527 nm), support the hypothesis that cryptochrome signalling is activated in the presence of this portion of the spectra whereas *phyA* and *phyB* seedlings did not have a circadian phenotype (Figure S3). It has previously been proposed that cryptochrome green light sensitivity either arises from residual sensitivity of the bound flavin chromophore at wavelengths longer than 500 nm (Ahmad et al., 2002; Ahmad, Lin, & Cashmore, 1995), or that irradiated cryptochromes absorb green light as part of their photocycle (Banerjee et al., 2007; Bouly et al., 2007). Our work comparing dark‐ and light‐adapted seedlings (Figure 3) suggests that irradiated cryptochromes contribute to the integration of green light signals into the circadian system, although the photochemistry underlying this phenotype remains to be investigated.

### 4.2 Exogenous sucrose masks the contribution of cryptochromes to circadian FRP under constant green light

Interpretation of light signalling into the circadian system is complicated by the clock's response to metabolites derived from photosynthesis (Frank et al., 2018; Haydon et al., 2013; Haydon et al., 2017; Philippou et al., 2019). In order to assess the contribution of photosynthesis towards circadian rhythmicity under constant green light, we assessed circadian rhythms in the presence of CO_2_‐depleted air (Figures 2d and 4). *cry1* seedlings retained an extended circadian FRP in CO_2_‐depleted conditions, although *cry2* seedlings were indistinguishable from wild type plants in a reduced CO_2_ environment. Such data suggests that cry1 acts in parallel to photosynthate‐derived signals to regulate circadian FRP under constant green light.

Exogenous sucrose has previously been used to saturate plants' circadian responses to photoassimilates (Frank et al., 2018; Haydon et al., 2013; Haydon et al., 2017; Philippou et al., 2019). Interestingly, exogenous sucrose was sufficient to mask the *cry1* circadian defect under constant green light but not constant blue light (Figures 4c and S4b). Although additional work remains to fully understand the interaction between cryptochrome and sucrose signalling, the addition of exogenous sucrose has been previously reported to mask the contribution of ethylene to circadian timing, highlighting the substantive role exogenous sucrose can have upon circadian rhythms (Haydon et al., 2017).

### 4.3 Cryptochrome signalling into the circadian system is distinct under either blue light and green light

Although cryptochromes contribute to both blue‐ and green‐light signalling pathways into the circadian system (Figures 3, 4, and 5), the consequences of *cry1* and *cry2* mutation are different for each lighting regime. Under constant blue light, *cry1* plants have a greatly reduced amplitude, with rhythms of *CCA1* and *GI* transcript accumulation trending towards arrhythmia (Figures 5a–c and S6a). These data are comparable with work showing that expression of *SIG5* is greatly reduced in *cry1* and *cry1cry2* plants under constant blue light (Belbin et al., 2016) but differ from earlier reports that included sucrose as a media additive (Devlin & Kay, 2000). By contrast, loss of cryptochrome function under green light causes an extension of circadian period without an associated loss of amplitude that is masked by the addition of supplemental sucrose (Figures 3, 4, 5, and S6b). These data suggest either that cryptochromes have distinct roles in circadian responses to blue or green light or that additional photoreceptors, such as phytochromes, additively contribute to circadian perception of green light.

Although it is difficult to directly compare different lighting regimes, we were interested to note that a combination of blue and green light was able to maintain the amplitude of bioluminescence in *cry1* seedlings with an extended circadian FRP phenotype (Figure 6c–e). Such data emphasize the ability of plants to perceive and integrate information from across the light spectrum to respond to prevailing environmental conditions. Previous work has identified physical interactions between cryptochromes and phytochromes, as well as between the signalling cascades induced by these photoreceptors (Hughes, Vrana, Song, & Tucker, 2012; Mas, Devlin, Panda, & Kay, 2000; Pedmale et al., 2016; Wang et al., 2018). It will consequently be of great interest to determine how phytochromes and cryptochromes interact to appropriately respond to green light as part of plants' complex response to natural illumination.

## Supporting information


**Figure S1.** **Circadian responses to very low fluences of blue light.** Waveforms of luciferase bioluminescence from wild type seedlings transformed with a *CCA1::LUC2* reporter. Seedlings were entrained for 6 days before transfer to constant blue light with a fluence of either 1 or 20 μmol m^‐2^ s^‐1^. Error bars indicate SEM and are shown every 10 hours for clarity, n > 20.

**Figure S2.** **Response of *TOC1::LUC* to increasing fluence rates of green light (a)** Waveforms of luciferase bioluminescence rhythms of wildtype *TOC1::LUC* seedlings under either constant darkness or increasing fluence rates of constant green light. Plants were entrained under 12:12 light:dark cycles for six days before transfer to constant conditions with the indicated fluence rate of green light. **(b)** Relative amplitude of circadian rhythms of luciferase bioluminescence presented in (a). **(c)** Circadian free running period estimates of data presented in (a). **(d)** Bioluminescence waveforms of wildtype *TOC1::LUC* seedlings imaged under diel cycles of 16 μmol m^‐2^ s^‐1^ green light. An extended dark period was introduced on the second day of imaging to examine entrainment of the circadian system to green light irradiation. Green bands indicate periods of green light whereas grey bands indicate periods of darkness. Error bars indicate SEM and in (a) and (d) are presented once every 10 hours for clarity, n > 10.

**Figure S3.** **Circadian green light responses in *phytochrome* mutants. (a + b)** Waveforms of luciferase bioluminescence in *CCR2::LUC* and *phyB‐9 CCR2::LUC* **(b)** Scatter plot of data shown in (a) comparing circadian free‐running period with relative amplitude error, a measure of rhythmic robustness (a perfect cosine wave having a value of 0), as calculated by Fourier fast transform‐nonlinear least squares. **(c + d)** Waveforms of luciferase bioluminescence in *CCA1::LUC2* and *phya‐211 CCA1::LUC2* **(d)** Scatter plot of data shown in (c) comparing circadian free‐running period with relative amplitude error. Seedlings were entrained for 6 days before transfer to 16 μmol m^‐2^ s^‐1^ of constant green light. Error bars indicate SEM and in (a) and (c) are shown every 10 hours for clarity, n > 20.

**Figure S4.** **Circadian rhythms of *cryptochrome* seedlings under constant blue light (a)** Waveforms of luciferase bioluminescence in wildtype *CCA1::LUC2* seedlings imaged under constant blue light in a CO_2_‐depleted environment. **(b)** Circadian free running period of light‐adapted plants transferred to constant blue light in the presence of exogenous sucrose or in a CO_2_‐depleted atmosphere. **(c)** Amplitude of circadian rhythms described in (b). Plants were grown on either MS plates or MS plates supplemented with 3% (w/v) sucrose. Seedlings were entrained for 6 days before transfer to 20 μmol m^‐2^ s^‐1^ constant blue light. Error bars indicate SEM and are shown every 10 hours for clarity, n > 10.

**Figure S5.** **Low amplitude rhythms of luciferase expression in *cry1* mutants.** Waveforms of luciferase bioluminescence from *cry1* seedlings are plotted on a separate axis to wild type *CCA1::LUC2*. Error bars indicate SEM and are shown every 10 hours for clarity. Data is replotted from Figure 5A.

**Figure S6.** **Transcript accumulation of *GIGANTEA* under either constant blue or constant green light.** Daily expression patterns of *GIGANTEA* in wild type, *cry1*, *cry2*, and *cry1cry2* seedlings transferred to 20 μmol m^‐2^ s^‐1^ of constant blue (a) or constant green light (b) after 12 days of entrainment. Data are the average of 3 independent experiments, error bars indicate standard error of the mean.

**Table S1.** Oligos used in this study.
