# Supplementary material for: Cryptochromes integrate green light signals into the circadian system
Source: Plant Cell Environ. 2019 Aug 27;43(1):16–27. doi: 10.1111/pce.13643 (PMC6973147; doi:10.1111/pce.13643)
Supplement: Supplementary file 1 — Figure S1. Circadian responses to very low fluences of blue light. Waveforms of luciferase bioluminescence from wild type seedlings transformed with a CCA1::LUC2 reporter. Seedlings were entrained for 6 days before transfer to constant blue light with a fluence of either 1 or 20 μmol m‐2 s‐1. Error bars indicate SEM and are shown every 10 hours for clarity, n > 20. [file PCE-43-16-s001.pdf]

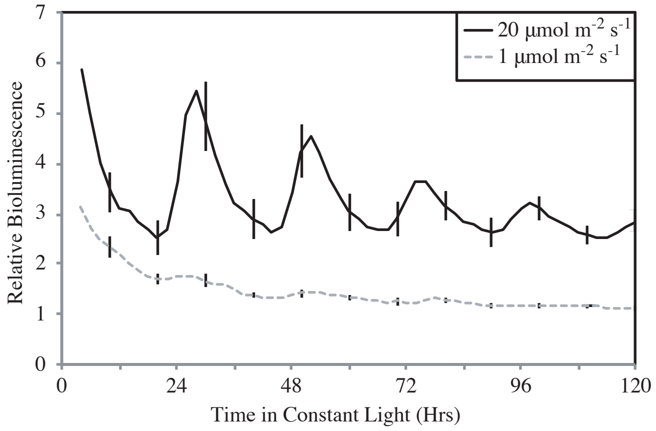

Supplemental Figure 1. Circadian responses to very low fluence rates of blue light. Waveforms of luciferase bioluminescence from wild type seedlings transformed with a *CCA1::LUC2* reporter. Seedlings were entrained for 6 days before transfer to constant blue light with a fluence of either 1 or 20 μmol m^{-2} s^{-1}. Error bars indicate SEM and are shown every 10 hours for clarity, n>20.
