# Supplementary material for: Cryptochromes integrate green light signals into the circadian system
Source: Plant Cell Environ. 2019 Aug 27;43(1):16–27. doi: 10.1111/pce.13643 (PMC6973147; doi:10.1111/pce.13643)
Supplement: Supplementary file 2 — Figure S2. Response of TOC1::LUC to increasing fluence rates of green light (a) Waveforms of luciferase bioluminescence rhythms of wildtype TOC1::LUC seedlings under either constant darkness or increasing fluence rates of constant green light. Plants were entrained under 12:12 light:dark cycles for six days before transfer to constant conditions with the indicated fluence rate of green light. (b) Relative amplitude of circadian rhythms of luciferase bioluminescence presented in (a). (c) Circadian free running period estimates of data presented in (a). (d) Bioluminescence waveforms of wildtype TOC1::LUC seedlings imaged under diel cycles of 16 μmol m‐2 s‐1 green light. An extended dark period was introduced on the second day of imaging to examine entrainment of the circadian system to green light irradiation. Green bands indicate periods of green light whereas grey bands indicate periods of darkness. Error bars indicate SEM and in (a) and (d) are presented once every 10 hours for clarity, n > 10. [file PCE-43-16-s002.pdf]

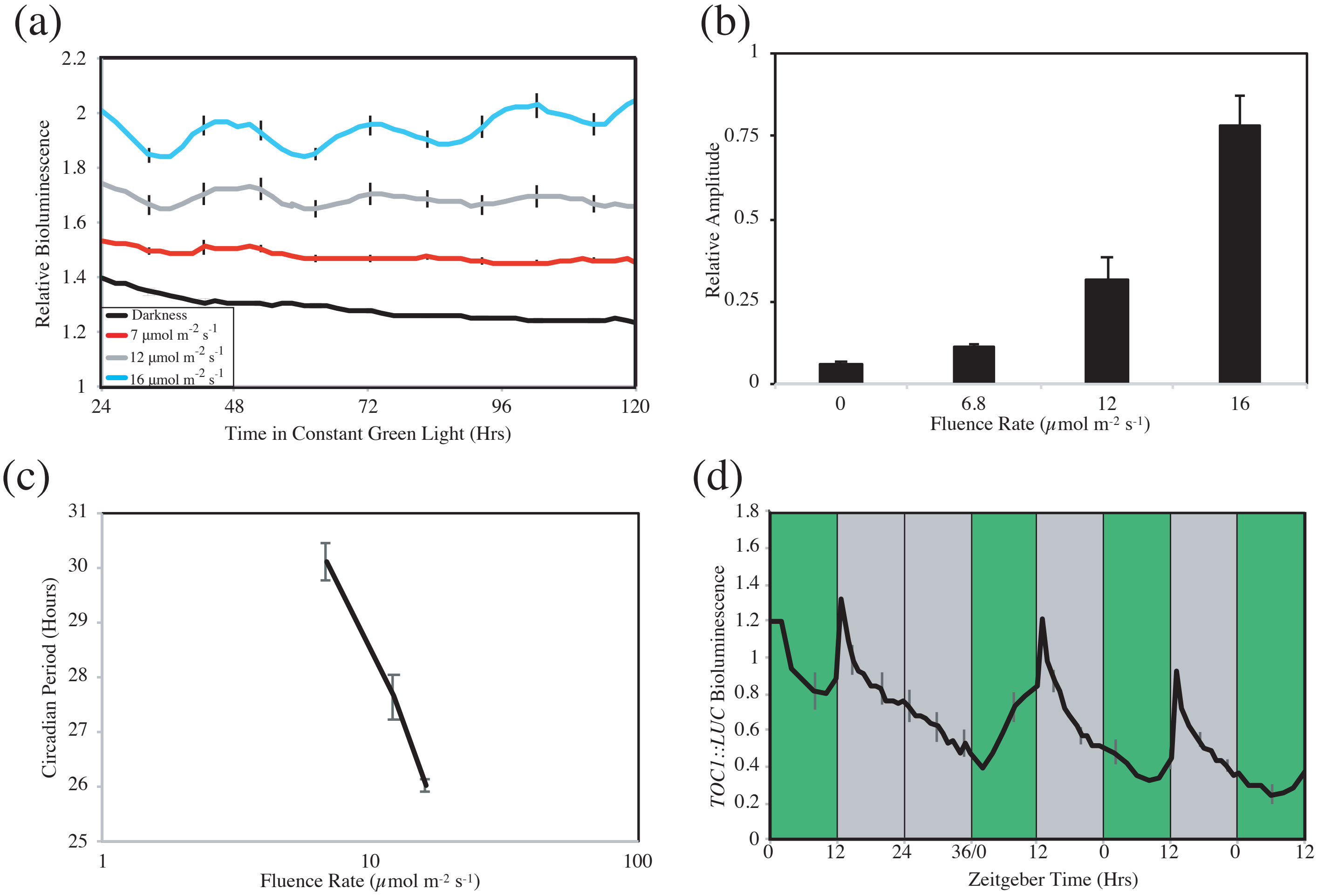

Supplemental Figure 2. Response of *TOC1::LUC* to increasing fluence rates of green light. (a) Waveforms of luciferase bioluminescence rhythms of wildtype *TOC1::LUC* seedlings under either constant darkness or increasing fluence rates of constant green light. Plants were entrained under 12:12 light:dark cycles for six days before transfer to constant conditions with the indicated fluence rate of green light. **(b)** Relative amplitude of circadian rhythms of luciferase bioluminescence presented in (a). **(c)** Circadian free running period estimates of data presented in (a). **(d)** Bioluminescence waveforms of wildtype *TOC1::LUC* seedlings imaged under diel cycles of 16 μmol m^{-2} s^{-1} green light. An extended dark period was introduced on the second day of imaging to examine entrainment of the circadian system to green light irradiation. Green bands indicate periods of green light whereas grey bands indicate periods of darkness. Error bars indicate SEM and in (a) and (d) are presented once every 10 hours for clarity, n>10.
