# Supplementary material for: Cryptochromes integrate green light signals into the circadian system
Source: Plant Cell Environ. 2019 Aug 27;43(1):16–27. doi: 10.1111/pce.13643 (PMC6973147; doi:10.1111/pce.13643)
Supplement: Supplementary file 3 — Figure S3. Circadian green light responses in phytochrome mutants. (a + b) Waveforms of luciferase bioluminescence in CCR2::LUC and phyB‐9 CCR2::LUC (b) Scatter plot of data shown in (a) comparing circadian free‐running period with relative amplitude error, a measure of rhythmic robustness (a perfect cosine wave having a value of 0), as calculated by Fourier fast transform‐nonlinear least squares. (c + d) Waveforms of luciferase bioluminescence in CCA1::LUC2 and phya‐211 CCA1::LUC2 (d) Scatter plot of data shown in (c) comparing circadian free‐running period with relative amplitude error. Seedlings were entrained for 6 days before transfer to 16 μmol m‐2 s‐1 of constant green light. Error bars indicate SEM and in (a) and (c) are shown every 10 hours for clarity, n > 20. [file PCE-43-16-s003.pdf]

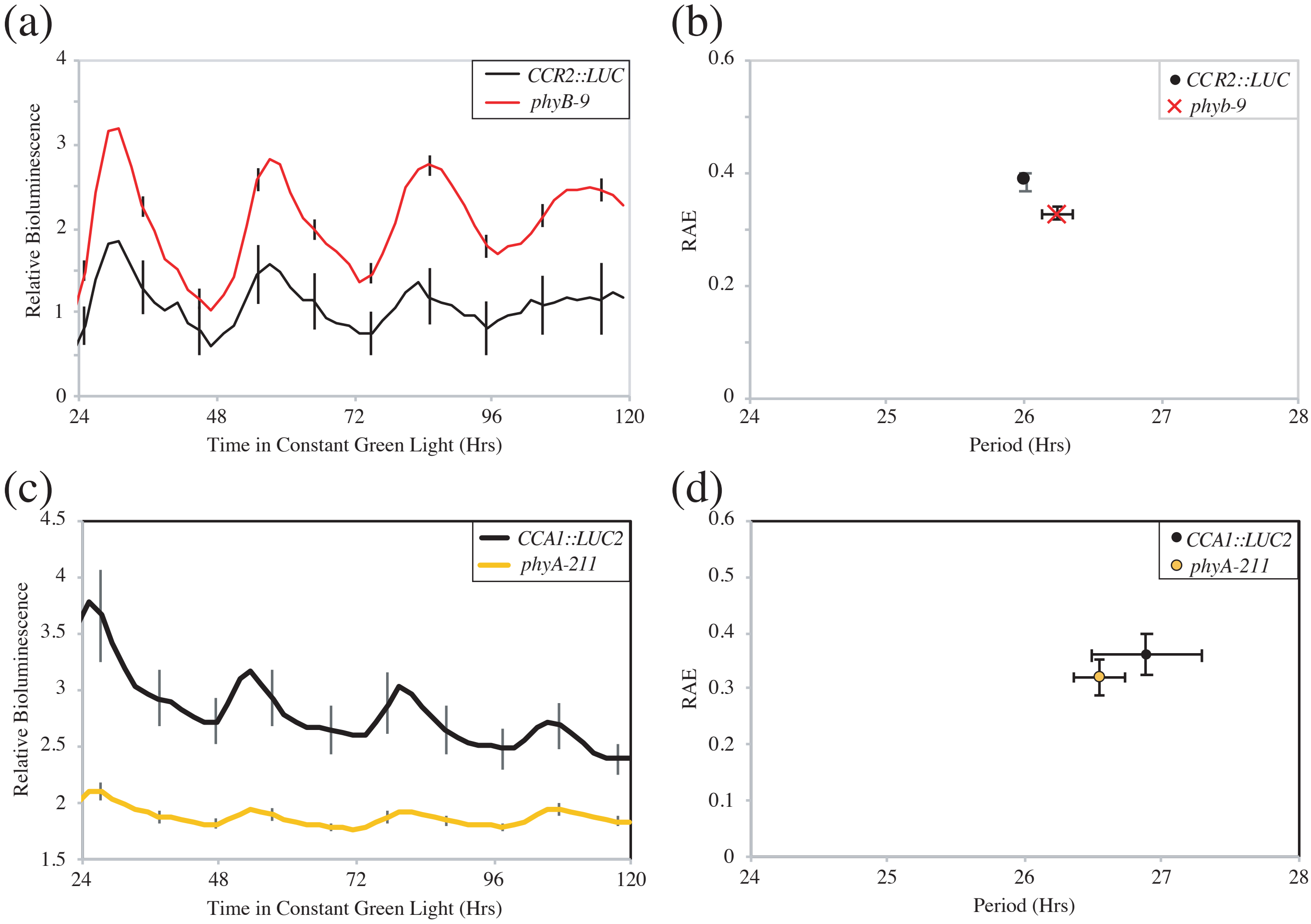

Supplemental Figure 3. Circadian green light responses in *phytochrome* mutants. (a) Waveforms of luciferase bioluminescence in wild type *CCR2::LUC* and *phyB-9 CCR2::LUC* seedlings under 16 µmol m^{-2} s^{-1} of constant green light. **(b)** Scatter plot of data shown in (a) comparing circadian free-running period with relative amplitude error, a measure of rhythmic robustness (a perfect cosine wave having a value of 0), as calculated by Fourier fast transform-nonlinear least squares. **(c)** Waveforms of luciferase bioluminescence in wild type *CCA1::LUC2* and *phyA-211 CCA1::LUC2* seedlings under 16 µmol m^{-2} s^{-1} of constant green light. **(d)** Scatter plot of data shown in (c) comparing circadian free-running period with RAE. Seedlings were entrained for 6 days before transfer to constant conditions. Error bars indicate SEM, n>20.
