# Supplementary material for: Cryptochromes integrate green light signals into the circadian system
Source: Plant Cell Environ. 2019 Aug 27;43(1):16–27. doi: 10.1111/pce.13643 (PMC6973147; doi:10.1111/pce.13643)
Supplement: Supplementary file 4 — Figure S4. Circadian rhythms of cryptochrome seedlings under constant blue light (a) Waveforms of luciferase bioluminescence in wildtype CCA1::LUC2 seedlings imaged under constant blue light in a CO2‐depleted environment. (b) Circadian free running period of light‐adapted plants transferred to constant blue light in the presence of exogenous sucrose or in a CO2‐depleted atmosphere. (c) Amplitude of circadian rhythms described in (b). Plants were grown on either MS plates or MS plates supplemented with 3% (w/v) sucrose. Seedlings were entrained for 6 days before transfer to 20 μmol m‐2 s‐1 constant blue light. Error bars indicate SEM and are shown every 10 hours for clarity, n > 10. [file PCE-43-16-s004.pdf]

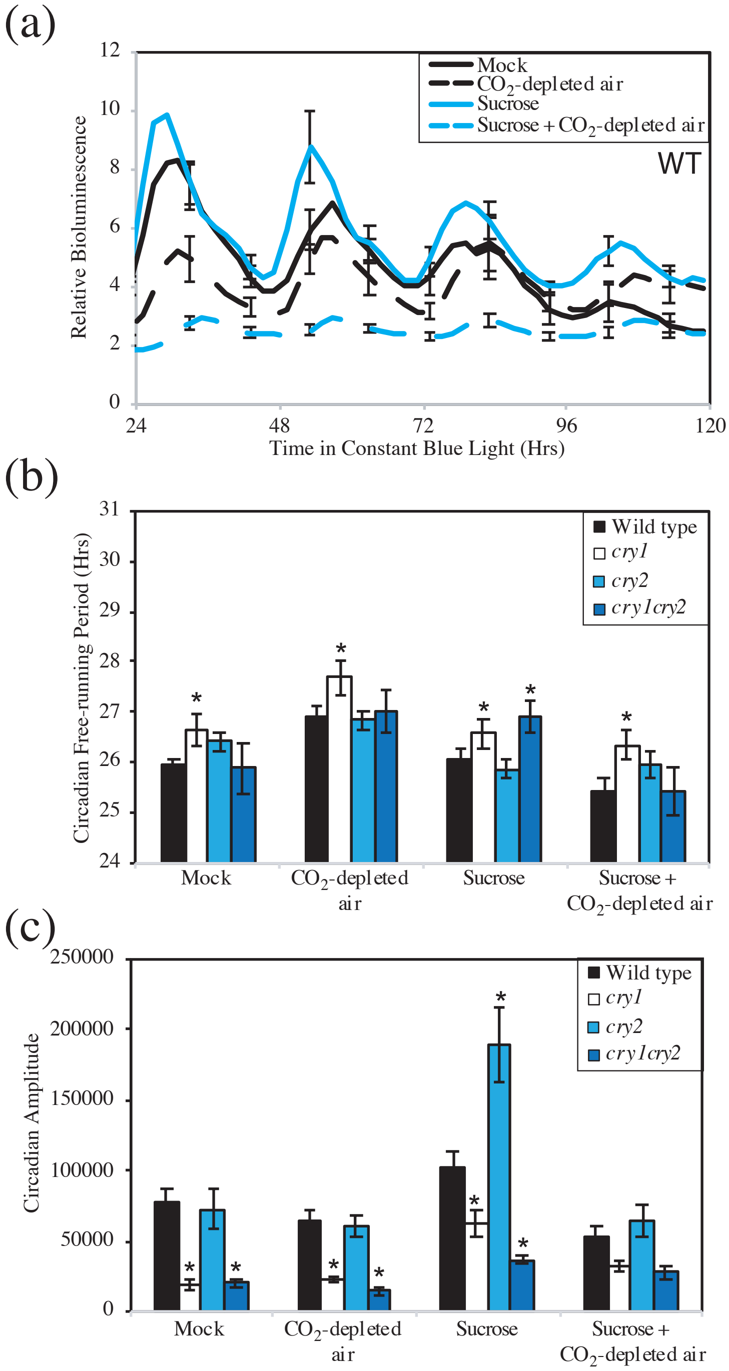

Supplemental Figure 4. Circadian rhythms of *cryptochrome* seedlings under constant blue light (a) Waveforms of luciferase bioluminescence in wildtype *CCA1::LUC2* seedlings imaged under constant blue light in a CO_2-depleted environment. **(b)** Circadian free running period of light-adapted plants transferred to constant blue light in the presence of exogenous sucrose or in a CO2 depleted atmosphere. **(c)** (c) Amplitude of circadian rhythms described in (b). Plants were grown on either MS plates or MS plates supplemented with 3% (w/v) sucrose. Seedlings were entrained for 6 days before transfer to 20 µmol m^{-2} s^{-1} of constant blue light. Error bars indicate SEM and are shown every 10 hours for clarity, n>10.
