# Supplementary material for: Cryptochromes integrate green light signals into the circadian system
Source: Plant Cell Environ. 2019 Aug 27;43(1):16–27. doi: 10.1111/pce.13643 (PMC6973147; doi:10.1111/pce.13643)
Supplement: Supplementary file 5 — Figure S5. Low amplitude rhythms of luciferase expression in cry1 mutants. Waveforms of luciferase bioluminescence from cry1 seedlings are plotted on a separate axis to wild type CCA1::LUC2. Error bars indicate SEM and are shown every 10 hours for clarity. Data is replotted from Figure 5A. [file PCE-43-16-s005.pdf]

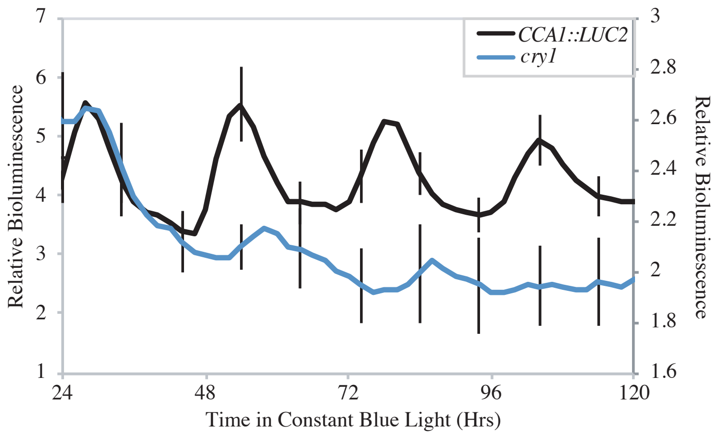

Supplemental Figure 5. Low amplitude rhythms of luciferase expression in *cry1* mutants. Waveforms of luciferase bioluminescence from *cry1* seedlings plotted on a separate axis to wild type *CCA1::LUC2*. Error bars indicate SEM and are shown every 10 hours for clarity. Data is replotted from Figure 5A.
