# Supplementary material for: Cryptochromes integrate green light signals into the circadian system
Source: Plant Cell Environ. 2019 Aug 27;43(1):16–27. doi: 10.1111/pce.13643 (PMC6973147; doi:10.1111/pce.13643)
Supplement: Supplementary file 6 — Figure S6. Transcript accumulation of GIGANTEA under either constant blue or constant green light. Daily expression patterns of GIGANTEA in wild type, cry1, cry2, and cry1cry2 seedlings transferred to 20 μmol m‐2 s‐1 of constant blue (a) or constant green light (b) after 12 days of entrainment. Data are the average of 3 independent experiments, error bars indicate standard error of the mean. [file PCE-43-16-s006.pdf]

(a)

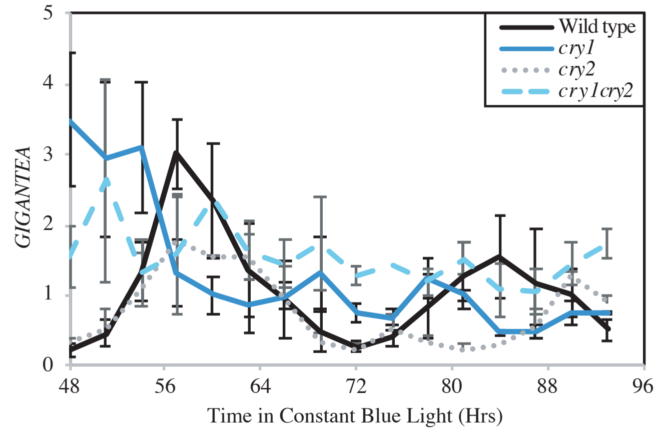

(b)

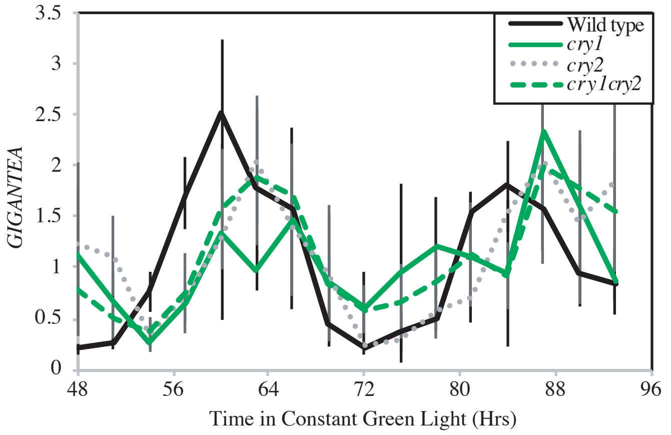

Supplemental Figure 6. Transcript accumulation of *GIGANTEA* under either constant blue or constant green light. Steady state abundance of *GIGANTEA* in wild type, *cry1, cry2*, and *cry1cry2* seedlings transferred to 20 µmol m^{-2} s^{-1} of constant blue **(a)** or constant green light **(b)** after 12 days of entrainment. Data are the average of 3 independent experiments, error bars indicate standard error of the mean.
