# Supplementary material for: Cryptochromes integrate green light signals into the circadian system
Source: Plant Cell Environ. 2019 Aug 27;43(1):16–27. doi: 10.1111/pce.13643 (PMC6973147; doi:10.1111/pce.13643)
Supplement: Supplementary file 7 — Table S1. Oligos used in this study. [file PCE-43-16-s007.pdf]

Oligo	Sequence	Reference
CCA1 qPCR F	CAGCTCCAATATAACCGATCCAT	Mockler *et al*. 2004
CCA1 qPCR R	CAATTCGACCCTCGTCAGACA	Mockler *et al*. 2004
GIGANTEA qPCR F	ACTAGCAGTGGTCGACGGTTTATC	Mizuno *et al*. 2014
GIGANTEA qPCR R	GCTGGTAGACGACACTTCAATAGATT	Mizuno *et al*. 2014
APX3 qPCR F	GCCGTGAGCTCCGTTCTCT	Nusinow *et al*. 2011
APX3 qPCR R	TCGTGCCATGCCAATCG	Nusinow *et al*. 2011
IPP2 qPCR F	GTATGAGTTGCTTCTGGAGCAAAG	Nusinow *et al*. 2011
IPP2 qPCR R	GAGGATGGCTGCAACAAGTGT	Nusinow *et al*. 2011
At1g11910 qPCR F	CTCCAGAAGAGTATGTTCTGAAAG	Nusinow *et al*. 2011
At1g11910 qPCR R	TCCCAAGATCCAGAGAGGTC	Nusinow *et al*. 2011

Supplemental Table 1. Oligos used in this study.
